# Phenotypic Characterization of Circulating Lung Cancer Cells for Clinically Actionable Targets

**DOI:** 10.3390/cancers11030380

**Published:** 2019-03-18

**Authors:** Arutha Kulasinghe, Joanna Kapeleris, Carolina Cooper, Majid Ebrahimi Warkiani, Kenneth O’Byrne, Chamindie Punyadeera

**Affiliations:** 1 The School of Biomedical Sciences, Room 603D, Institute of Health and Biomedical Innovation, Queensland University of Technology, 60 Musk Avenue, Kelvin Grove, QLD 4059, Australia; arutha.kulasinghe@qut.edu.au (A.K.); joanna.kapeleris@qut.edu.au (J.K.); 2 Translational Research Institute, Brisbane, QLD 4102, Australia; k.obyrne@qut.edu.au; 3 Department of Anatomical Pathology, Pathology Queensland, Brisbane, QLD 4006, Australia; Caroline.Cooper@health.qld.gov.au; 4 Princess Alexandra Hospital, Woolloongabba, QLD 4102, Australia; 5 The School of Biomedical Engineering, University of Technology Sydney, Ultimo, NSW 2007, Australia; Majid.Warkiani@uts.edu.au; 6 Department of Biomedical Engineering, Institute of Molecular Medicine, Sechenov First Moscow State Medical University, Moscow 119991, Russia

**Keywords:** liquid biopsy, circulating tumour cells, non-small cell lung cancer, actionable mutations

## Abstract

Objectives: In non-small cell lung cancers (NSCLC), tumour biopsy can often be an invasive procedure. The development of a non-invasive methodology to study genetic changes via circulating tumour cells (CTCs) is an appealing concept. Whilst CTCs typically remain as rare cells, improvements in epitope-independent CTC isolation techniques has given rise to a greater capture of CTCs. In this cross sectional study, we demonstrate the capture and characterization of NSCLC CTCs for the clinically actionable markers epidermal growth factor receptor (EGFR) alterations, anaplastic lymphoma kinase (ALK) rearrangements and programmed death ligand-1 (PD-L1) expression. The study identified CTCs/CTC clusters in 26/35 Stage IV NSCLC patients, and subsequently characterized the CTCs for EGFR mutation, ALK status and PD-L1 status. This pilot study demonstrates the potential of a non-invasive fluid biopsy to determine clinically relevant biomarkers in NSCLC.

## 1. Introduction

Lung cancer is the leading cause of cancer-related mortality worldwide, with non-small-cell lung cancers (NSCLCs) accounting for approximately 85% of all lung cancer cases [1,2]. NSCLC are often diagnosed at an advanced stage of disease, leading to poor survival rates, with a 5-year survival rate of 20% [1]. Treatment options for lung cancer include chemotherapy and the use of targeted agents for specific molecular targets or pathways. Tyrosine kinase inhibitors (TKIs) have shown benefit in patients carrying EGFR gene mutations. Moreover, tumours that contain anaplastic lymphoma kinase (ALK) fusion oncogene rearrangements, usually associated with never smokers, a younger age group and adenocarcinoma with signet or acinar histology, are sensitive to ALK-targeted therapies [3]. Therefore the assessment of the molecular profile of a patient’s tumour identifies clinically relevant patient subgroups in which personalized targeted treatment is most appropriate. Treatment options remain limited for locally advanced or metastatic NSCLC patients. Recently immunotherapies and immune checkpoint blockade of the interactions between programmed cell death protein 1 (PD-1) and its tissue ligand (PD-L1), using monoclonal antibodies, has shown significant and durable benefits compared to standard therapy in NSCLC [4,5]. Due to the immune related adverse effects and cost of immunotherapies, it is paramount to select patients most likely to benefit from the therapy [6]. Currently, PD-L1 tumour tissue scoring is used clinically to determine likelihood of response to therapy, however this has shown to have limitations in determining cutoff for PD-L1 positivity reflecting the dynamic nature of PD-L1 as a biomarker [7,8]. Therefore, alternative sources of tumour tissue, more reflective of the disease burden are required to predict responders for immunotherapy [9].

Circulating tumour cells (CTCs) are considered to be the transient metastatic cells responsible for metastasis [10,11]. CTCs are indicators of residual disease and are associated with an increased risk of metastasis [12]. The utility of CTCs has been termed “fluid phase/liquid biopsy” to assess quantitative and qualitative properties of the cancer non-invasively [13,14,15,16]. Whilst the FDA-approved CTC enrichment and detection platform CellSearch^®^ (Menarini Silicon Biosystems Inc, Huntingdon Valley, PA, USA) demonstrated the clinical utility of CTCs in prostate, colorectal and breast cancers [17], the data in NSCLC has remained limited [18]. Moreover, recent reports highlight the complexities with CTC capture due to low EpCAM expression and CTC heterogeneity [19,20]. One strategy to capture CTCs from the peripheral blood has been the use of microfluidic technologies, which has, in comparison to the CellSearch^®^, demonstrated to capture a greater proportion of CTCs from circulation [21,22]. In this study we present preliminary data on the use of spiral microfluidic technology to isolate CTC from 35 NSCLC patients. The technology separates out the CTCs utilizing hydrodynamic forces present in curvilinear microchannels for size-based sorting. Putative CTCs were evaluated for the presence of the clinically relevant markers EGFR exon 19 deletion, ALK rearrangements and PD-L1 status using immunocytochemistry and DNA FISH.

## 2. Results

### 2.1. Patient Characteristics

A total of 35 stage IV NSCLC patients and 10 normal healthy controls were included in this study. Patient characteristics are shown in Table 1. The median age was 63 years (range 34–84); including men (*n* = 21), woman (*n* = 14). The histological classifications for the patient cohort were adenocarcinoma (85.7%) and squamous cell carcinoma (14.3%). The clinicopathological patient findings are presented on (Table 1).

### 2.2. CTC Enrichment and Characterization

**Figure 1:**
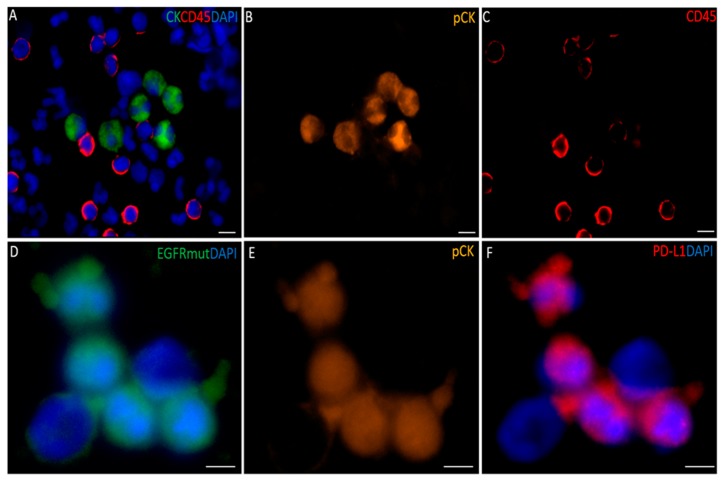
(**A**) Composite image of a circulating tumour cell (CTC) enriched sample from a non-small cell lung cancer (NSCLC) patient stained for pan-cytokeratin (green), common leukocyte marker CD45 (red), and nuclear stain DAPI (blue). (**B**) Individual pan-cytokeratin stain. (**C**) Individual CD45 stain. (**D**) Composite image of CTCs stained with EGFR E746-A750 deletion specific antibody and DAPI. (**E**) pan cytokeratin in the EGFR mut cells (**F**) Image of a CTCs stained with PD-L1 and DAPI. Cells in images (**D**,**E**) were negative for CD45. Scale bar represents 10 µm.

**Figure 2:**
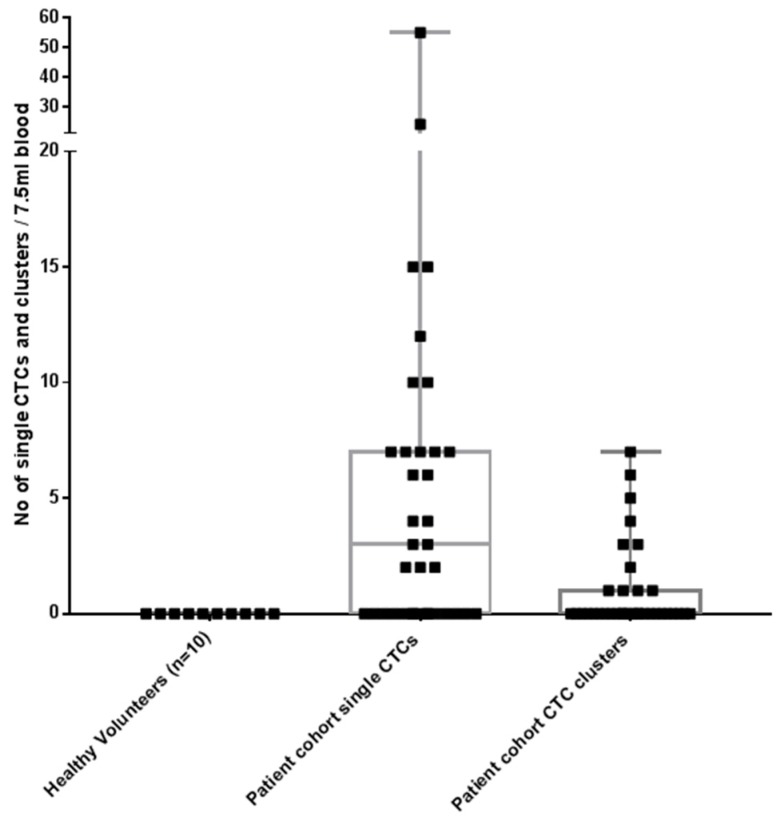
Circulating tumour cells (CTCs) in patients with non-small cell lung cancer (NSCLC) and normal healthy volunteers. CTCs were identified in 26/35 patients (either single CTCs/CTC clusters). No CTC-like events were observed in the normal healthy volunteer samples. CTCs defined as pan-cytokeratin^+^, CD45^−^, DAPI^+^.

**Figure 3:**
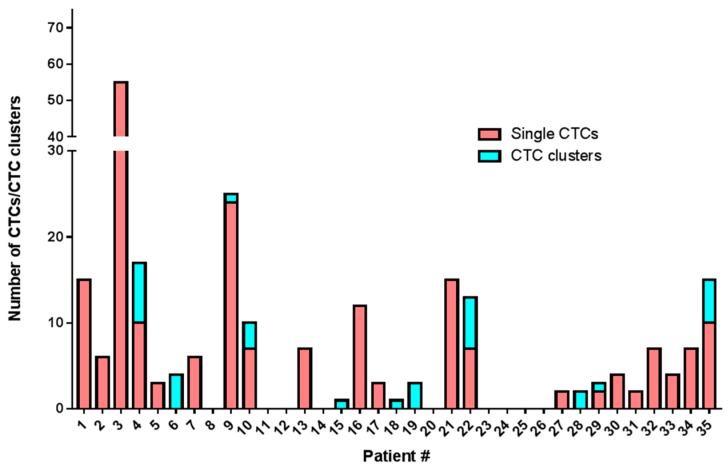
Distribution of CTCs (single-red) and CTC clusters (blue) in the NSCLC patient cohort.

All blood samples had two rounds of enrichment through the spiral chip and CTCs enumerated and characterized. Putative single CTCs and CTC clusters were identified as pan-cytokeratin^+^, DAPI^+^ and CD45^−^ (Figure 1). White blood cells were identified as CD45^+^ and DAPI^+^. The distribution of CTC types in the sample cohort is shown in (Figure 2) and the breakdown of the numbers of CTCs is illustrated in (Figure 3). Cells pan-cytokeratin^+^ and DAPI^+^ were stained for EGFR exon 19 deletion and PD-L1 expression. CTC-like events were not observed in the normal healthy volunteer samples. Single CTCs were identified in 21/35 samples (range 1–55 CTCs/7.5 mL blood) and CTC clusters in 11/35 samples (1–7 CTC clusters/7.5 mL), with single CTCs also found in 6/11 of the samples exhibiting CTC clusters. The numbers of CTCs are comparable to previously published studies in NSCLC [23,24].

**Figure 4:**
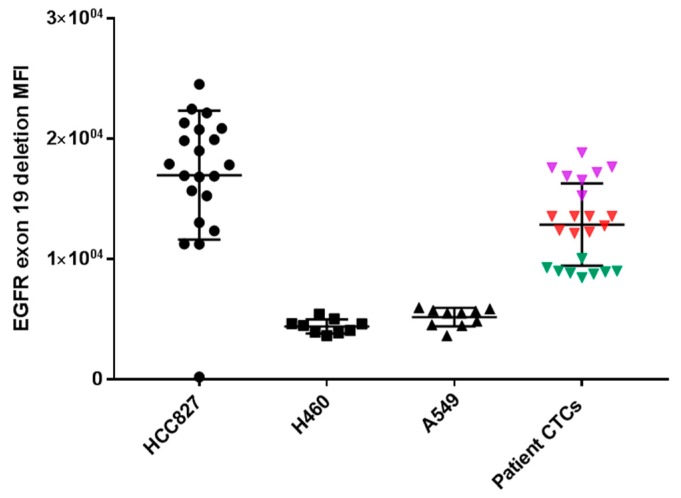
EGFR mutation status (exon 19 deletion) measured by mean fluorescence intensity (MFI) in NSCLC cell lines (HCC827; positive for exon 19 deletion), (H460 and A549; negative for exon19 deletion) and the patient CTC exon 19 deletion status. The individual colours represent individual CTCs within the same sample/patient.

**Figure 5:**
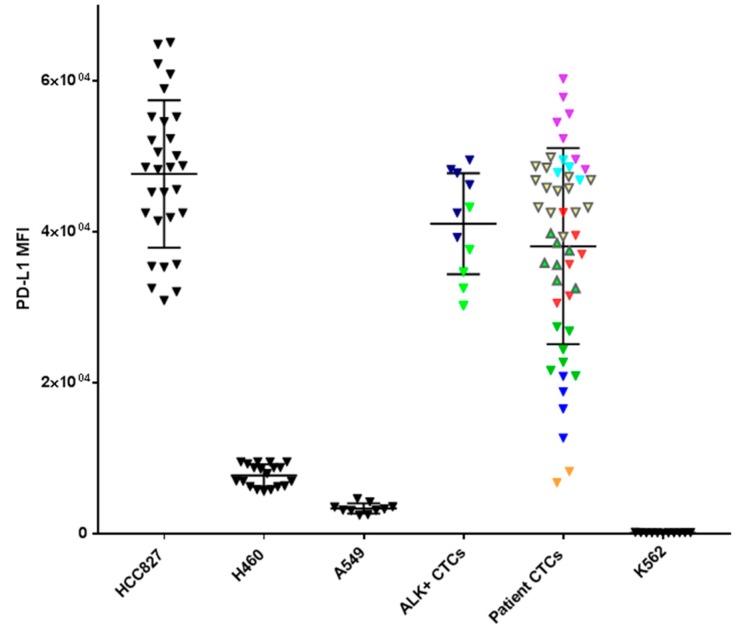
Programmed death ligand-1 (PD-L1) status measured by mean fluorescence Intensity (MFI) of NSCLC cell lines (HCC827, H460, A549), patient CTC samples (each colour representing a different patient sample and the individual data point CTC PD-L1 expression), ALK+ CTCs and K562 (negative control). The individual colours represent individual CTCs within the same sample.

**Figure 6:**
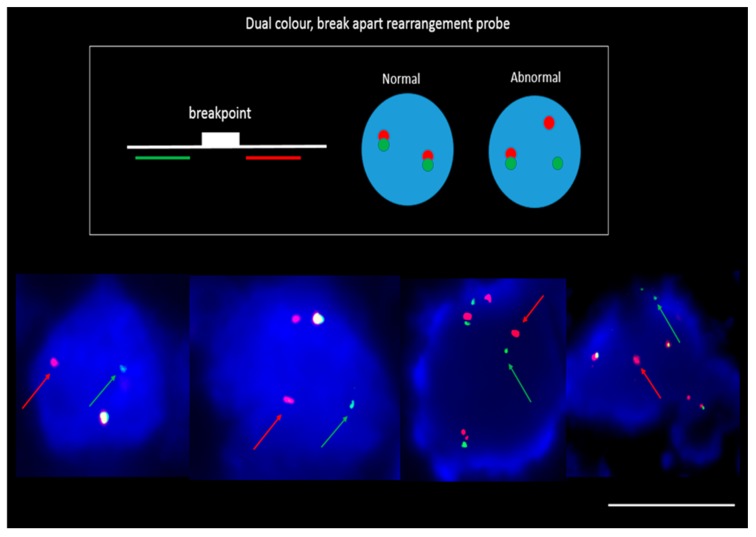
Molecular fluorescence in-situ hybridization (FISH) analysis on enriched CTCs with NSCLC. Cells were stained using Vysis Break Apart FISH probe and counterstained with DAPI. The red and green signals demonstrate a separation of the original fusion signal (arrows), indicating a rearrangement in the 2p23 ALK-gene locus. Scale bar represents 10 µm.

In three patients (Pt #1, #9 and #13) where EGFR exon 19 deletion was identified in the primary tissue by pathology DNA sequencing and matched patient bloods were taken for CTC assessment, CTCs stained positive with an EGFR exon 19 deletion specific antibody (EGFR E746-A750) (a minimum of one CTC with positive staining; 8/15 positive for Pt#1, 8/24 positive for Pt#9 and 7/7 positive for Pt#13). The intensity of patients’ CTC EGFR mutation staining was found to be comparable to that of a lung cell line having an EGFR exon 19 deletion—HCC827 (Figure 4). Five patients known to be EGFR del 19 negative were also stained and no immunoreactivity was seen. PD-L1 expression was evaluated in 18 patients found to have CTCs. In 10 patients PD-L1 expression, defined as the presence of at least one PD-L1 positive cell, was found. The expression was compared with PD-L1 high (HCC827) and low (A549, H460) NSCLC cell lines and a negative control (K562) which were used to comparatively measure PD-L1 expression. The NSCLC patient CTCs demonstrated a range of expression (low-high). Within each patient however, the CTC PD-L1 immunofluorosence intensity was comparable between cells forming a ‘cluster’ effect for the overall analysis (Figure 5). A further eight patients presented with no PD-L1 positive CTCs. In the three patients with EGFR exon-19 deletion, a mid-high range of PD-L1 expression was found (6/15 positive in Pt#1, 6/24 #9 and 7/7 in #13—Figure 4 and Figure 5). Of the four patient’s that had EML4-ALK positive tumours, two of the patient’s had detectable CTC counts (Pt #34, 35). EML4-ALK was found to be translocated in more than half the number of total CTCs per patient (5/7 CTCs in Pt #34 and 6/10 CTCs in Pt #35) (Figure 6). PD-L1 was found to be highly expressed in the ALK positive CTCs (Figure 5).

## 3. Discussion

In this prospective cross-sectional study, CTCs (single/clusters) were identified in 26/35 (74.3%) NSCLC patient samples, comprised of single CTCs and CTC clusters. Whilst the data on CTC clusters remains limited, they have been reported to have a higher metastatic potential compared to single CTCs in solid tumours [25]. CTC clusters may be able to traverse narrow capillaries, en route to distant sites [26] with recent studies highlighting that neutrophils escort CTCs [27,28,29]. The clinically important biomarkers EGFR del 19 mutants, ALK rearrangements and PD-L1 expression were evaluable in a subset of patient CTCs. The assessment of CTCs to determine EGFR mutation status, ALK rearrangements and PD-L1 status lends itself to the identification of patients for targeted therapies, be they oncogenic or immune-related, in patients where tumour biopsy tissue may not be available [30,31,32].

This is, to our knowledge, the first study to demonstrate EGFR mutation detection in CTCs using protein immunofluoresence. The antibody chosen has been demonstrated to be highly specific and good sensitivity in the detection of EGFR del 19 mutations in tissue samples [33]. However, there remains a lack of antibodies for other clinically relevant EGFR mutations such as EGFR L858R and T790M. Our data suggests that these antibodies may also prove effective in the CTC setting, giving positive immunofluorescence in the EGFR del 19 CTCs only as compared EGFR wild-type samples. With optimization and validation this approach holds promise for detecting such mutations in NSCLC patients particularly where tumour tissue is not available [34,35]. The technique is relatively inexpensive, has the potential to be developed into a simple and standardized assay that could prove useful as an initial screening tool in clinic and has a rapid turn-around time, results being available within 2–3 working days provided CTCs are isolated [36]. In ongoing work, we are currently evaluating the expansion of CTCs through ex-vivo culture for the testing of EGFR targeted therapies. This method has recently been described as having the potential to be used for genomic testing [37]. There remain a number of limitations in CTC research due to the low numbers of CTCs released into the blood of cancer patients at early stages of disease [38]. Moreover, CTCs represent primary and metastatic events and therefore are heterogeneous [15,39]. Therefore, CTCs would need to be used in conjunction with tissue and plasma tumoural data to provide the compliment of solid tumour and liquid biopsy [40,41].

The presence of ALK rearrangement on CTCs validates previously published studies [42,43]. Tumour tissue currently uses a threshold of 15% of ALK-rearranged cells for the diagnosis of an ALK-rearrangement positive tumour. Therefore cutoff values need to be established for CTCs. Pailler et al., have described that the number of ALK-rearranged CTCs per volume of blood be reported rather than the percentage of ALK-rearranged CTCs as a cutoff value for establishing the diagnosis of ALK rearrangements. In their study, four or more ALK-rearranged CTCs/mL of blood was established as diagnostic [43]. In the study by Ling Tan et al., a false positive cutoff for ALK break-apart probes was established (≤2 CTCs/1.88 mL blood) [44].

In a recent breast cancer study, Mazel et al., described the isolation and quantification of CTCs for PD-L1 [31]. We applied the same analysis to our patients and were likewise able to establish a scoring system relating CTC PD-L1 expression to that seen in cell lines. Whilst preliminary, this data may be used to develop a CTC PD-L1 immunoscore, to identify potential patients with high PD-L1^+^ CTCs for immunotherapy with single agent PD-1 targeted immunotherapy in the first line setting where tissue samples are not available [22,31]. Conversely, in patients with low PD-L1^+^ CTCs, an alternative therapeutic approach may be required [45,46]. 

In the future, based on our data, we plan to investigate the relationship between PD-L1 expression in CTCs and tumour tissue samples, linking these observations to expression in tissue samples and to objective outcomes in patients in terms of response rates and survival following treatment with immune checkpoint inhibitors. Our proposed future approach is underpinned by a number of recent studies [12,22,31,47]. In a report by Nicolazzo et al., patients receiving Nivolumab had PD-L1 expression in CTCs monitored longitudinally by CellSearch™. The persistence of PD-L1^+^ CTCs was found to be indicative of progressive disease whereas PD-L1^−^ cases demonstrated a clinical benefit [47]. Whilst the FDA approved CellSearch™ platform remains the gold standard, its inherent preselection for EpCAM-positive CTCs may not account for the heterogeneity observed in CTCs [48,49]. Therefore, paired studies, incorporating the CellSearch™ and label-free CTC technologies may be needed to identify the role of particular populations of CTCs and may be more informative of response to PD-1 and PD-L1 targeted immune checkpoint inhibitors [50,51,52]. More importantly, these PD-L1^+^ CTCs may mirror mechanisms of immune evasion and treatment resistance in NSCLC and need to be investigated further [47,53].

Of interest is the finding that PD-L1 expression in oncogene driven CTCs appears to be higher than that seen in wild type tumours. Similar findings have been reported in NSCLC cell line studies [54,55]. In the CTCs that were EGFR mutation positive, however, not all the cells were likewise PD-L1 positive. This could be due to inherent CTC heterogeneity found within circulation [56]. The observation is in contrast to most reports in tumour tissue that indicate that PD-L1 expression is lower in EGFR mutation positive cases that wild-type variants. Furthermore, oncogene driven tumors are less likely to respond to immune therapies than wild type disease, most likely related not only to PD-L1 expression levels but also to the lower tumour mutation burden in these cells [9,57]. Further work is required to understand the mechanisms behind PD-L1 expression in CTCs and the potential relationship between the expression and likely benefit from immunotherapy.

## 4. Materials and Methods

### 4.1. Patient Cohort

Ethics approval was obtained from the Metro South Health District Human Research Ethics Committee in accordance with the National Health and Medical Research Council’s guidelines (HREC/11/QPAH/331) to collect blood samples from the Princess Alexandra Hospital (PAH). All methods were performed in accordance with these ethical guidelines and regulations. This study has also been given institutional approval from the Queensland University of Technology human ethics committee (1100001420). Following written informed consent, 15–20 mL of blood samples were collected from advanced stage treatment naïve NSCLC patients presenting to the outpatient lung clinic.

### 4.2. Cell Lines and Culture

The cell lines H460 (ATCC^®^HTB-177™), HCC827 (ATCC^®^CRL-2868™), (A549 ATCC^®^CCL-185™) were a generous gift from Dr Mark Adams (QUT, Brisbane, Australia), and human chronic myelogenous leukemia K562 (ATCC^®^CCL-243) from Prof Maher Gandhi (UQDI, Brisbane, Australia). Cells were cultured under standard conditions in humidified incubators at 37 °C, 5% CO_2_ in RPMI-1640-Glutamax (Life Technologies, Inc., Foster, CA, USA) supplemented with 10% fetal bovine serum (FBS) and 1% Penicillin/Streptomycin (Pen/Strep). Cell line authenticity was routinely confirmed by Short Tandom Repeat (STR) profiling with the Stem Elite™ ID System (Promega, Madison, WI, USA) according to the manufacturer’s instructions. Cell lines were confirmed negative for mycoplasma infection by Hoechst staining and PCR [58].

### 4.3. Enrichment of Circulating Tumour Cells

CTCs were isolated using the spiral microfluidic technology as previously described [59,60]. Briefly, to reduce the cellular components passing through the microfluidic chip, 10 mL of whole blood, collected in K2EDTA tubes (BD-Plymouth, Plymouth, UK) was red blood cell lysed (Astral Scientific, Taren Point, Australia), and run through a slanted spiral chip using a syringe pump at 1.7 mL/min. The CTC output was collected and run back through the spiral chip to reduce the contaminating leukocytes in the final preparation. Two rounds of enrichment were used to have a 2–3 log fold reduction in white blood cells [21]. The final CTC output was collected and spun down at 300 g for 5 min.

### 4.4. Characterization of Circulating Tumour Cells

The enriched samples were cyto-centrifuged onto glass slides and CTCs identified by immunocytochemistry (ICC) using the CellSearch^®^ antibody cocktail (pan-Cytokeratin/CD45/DAPI; Menari-Silicon Biosystems, PA, USA). Briefly, the slides were air dried overnight and incubated with the combination of CellSearch^®^ (Menarini Silicon Biosystems Inc, PA, USA) reagents (20 µL staining reagent, 20 µL permeabilization buffer, 20 µL fixation buffer, 10 µL DAPI and 130 µL 1× PBS). The slide was incubated for 1.5 h at room temperature, washed three times in 1× PBS. Putative CTCs were further characterized for EGFR exon 19 deletion (1:100 dilution) (EGFR E746-A750, Cell Signaling, Beverly, MA, USA) and PD-L1 (1:200 dilution) (anti-PD-L1 (28-8)). The slides were mounted with Prolong Gold mounting medium (Molecular Probes, Eugene, OR, USA) to prevent photo-bleaching and preserve the fluorescent labelled molecules for long term storage, coverslipped and imaged using a Nikon Epifluorescence microscope (Nikon, Tokyo, Japan). CTCs were identified as intact, pan-cytokeratin^+^, DAPI^+^, CD45^−^ cells larger than 4 µm. The mean fluorescence intensity (MFI) was measured per CTC by determining the fluorescence intensity per CTC and subtracting this from the local background intensity. This was compared to the MFI of known NSCLC cancer cell lines and controls.

### 4.5. DNA Fluorescence In-Situ Hybridization (FISH)

CTC-enriched cyto-centrifuged samples were fixed in 4% paraformaldehyde and dehydrated via an ethanol series (70%, 85%, 96%). Slides were treated with RNAse (4 mg/mL) (Sigma-Aldrich, St. Louis, MO, USA) and DNA FISH carried out with EML4-ALK probe (Vysis LSI ALK break apart, Abbott, Lake Bluff, IL, USA) as previously described [61] and counterstained with DAPI. The cytospots was coverslipped and imaged on a Nikon Eclipse Ti inverted microscope fitted with a Nikon digital camera. The Nikon NIS Elements software was used for analysis. FISH parameters (z-stacking, distance between z-stacks and exposure time) were optimized for FISH signal identification. ALK status was assessed and scored independently by an experienced cytogeneticist.

### 4.6. Ethics Approval and Consent to Participate

Ethics approval was obtained from the Metro South Health District Human Research Ethics Committee in accordance with the National Health and Medical Research Council’s guidelines (HREC/11/QPAH/331) to collect blood samples from the Princess Alexandra Hospital (PAH). All methods were performed in accordance with the Declaration of Helsinki. This study has also been given institutional approval from the Queensland University of Technology human ethics committee (1100001420).

## 5. Conclusions

This study provides further proof of concept in the identification of clinically actionable markers in NSCLC CTCs. Further studies are warranted to validate these in a larger patient cohort and identify further markers of interest such as ROS1 and RET rearrangements, other EGFR mutants and in NRAS-, KRAS-mutated adenocarcinomas.

## Figures and Tables

**Table 1:** Clinicopathological findings. Numbers in the brackets represent the number of circulating tumour cells (CTCs) positive for that marker.

Pt#	Gender	Age	Lung Cancer Type	Stage	Subtype (Tissue)	Single CTC Enumeration (pCK/CD45/DAPI)/7.5 mL	CTC Cluster Enumeration (pCK/CD45/DAPI)/7.5 mL	Further CTC Characterization
1	F	60–65	NSCLC	IV	Adenocarcinoma EGFR mutation Deletion 19	15	0	EGFR mutation+ (8) PD-L1+ (6)
2	M	70–75	NSCLC	IV	Adenocarcinoma EGFR mutation Exon 21	6	0	PD-L1−
3	M	60–65	NSCLC	IV	Squamous cell carcinoma	55	0	PD-L1+ (14)
4	F	80–85	NSCLC	IV	Adenocarcinoma	10	7	PD-L1−
5	M	55–60	NSCLC	IV	Squamous cell carcinoma	3	0	PD-L1−
6	M	60–65	NSCLC	IV	Adenocarcinoma	0	4	
7	F	50–55	NSCLC	IV	Adenocarcinoma	6	0	PD-L1+ (4)
8	F	70–75	NSCLC	IV	Adenocarcinoma	0	0	
9	F	80–85	NSCLC	IV	Adenocarcinoma EGFR mutation Deletion 19	24	1	EGFR mutation+ (8) PD-L1+ (6)
10	F	75–80	NSCLC	IV	Adenocarcinoma	7	3	PD-L1-
11	M	60–65	NSCLC	IV	Adenocarcinoma ALK+	0	0	
12	M	60–65	NSCLC	IV	Adenocarcinoma	0	0	
13	M	45–50	NSCLC	IV	Adenocarcinoma EGFR mutation Deletion 19 T790 mutant	7	0	EGFR mutation+ (7) PD-L1+ (7)
14	M	45–50	NSCLC	IV	Adenocarcinoma ALK+	0	0	
15	M	67–70	NSCLC	IV	Adenocarcinoma	0	1	
16	M	60–65	NSCLC	IV	Adenocarcinoma	12	0	PD-L1−
17	M	40–45	NSCLC	IV	Adenocarcinoma	3	0	
18	M	65–70	NSCLC	IV	Adenocarcinoma	0	1	
19	F	65–70	NSCLC	IV	Adenocarcinoma	0	3	
20	M	65–70	NSCLC	IV	Squamous cell carcinoma	0	0	
21	F	50–55	NSCLC	IV	Adenocarcinoma KRAS mutant	15	0	PD-L1+ (8)
22	F	60–65	NSCLC	IV	Squamous cell carcinoma	7	6	PD-L1+ (4)
23	F	70–75	NSCLC	IV	Adenocarcinoma	0	0	
24	F	70–75	NSCLC	IV	Adenocarcinoma	0	0	
25	M	70–75	NSCLC	IV	Adenocarcinoma	0	0	
26	M	80–85	NSCLC	IV	Adenocarcinoma	0	0	
27	M	70–75	NSCLC	IV	Adenocarcinoma	2	0	PD-L1−
28	M	35–40	NSCLC	IV	Adenocarcinoma	0	2	
29	F	55–60	NSCLC	IV	Adenocarcinoma	2	1	PD-L1+ (2)
30	M	70–75	NSCLC	IV	Squamous cell carcinoma	4	0	
31	F	65–70	NSCLC	IV	Adenocarcinoma	2	0	PD-L1−
32	M	70–75	NSCLC	IV	Adenocarcinoma	7	0	
33	M	60–65	NSCLC	IV	Adenocarcinoma	4	0	PD-L1−
34	M	30–35	NSCLC	IV	Adenocarcinoma ALK+	7	0	ALK+ (5) PD-L1+ (6)
35	F	50–55	NSCLC	IV	Adenocarcinoma ALK+	10	5	ALK+ (6) PD-L1+ (5)

NSCLC: Non-small cell lung cancer; EGFR: Epidermal growth factor receptor; PD-L1: Programmed death ligand-1; ALK: Anaplastic lymphoma kinase.
